# Conservative Surgery

**Published:** 1861-07

**Authors:** H. Wanzer

**Affiliations:** Chicago, Ill.


### ARTICLE IV.

### CONSERVATIVE SURGERY.

A Case by H. WANZER, M. D., of Chicago, Ill.

The following case may serve to show how much may often be gained for the benefit of a patient, by not being in too much haste to amputate injured members.

M. B——, a boy aged fourteen years, was brought to my office, May 4, 1861. He had been incautiously manipulating with a circular saw, during which, the left hand became engaged, and the index and middle fingers nearly amputated, at the base of the first phalanges. The skin, fascia, bones, flexor tendons, and digital arteries were divided, so that the ends of the fingers could be easily turned over upon the dorsal surface, leaving only a connection of integument and cellular tissue. The ring and little fingers were injured, but not so severely.

But for the solicitation of one of my assistants, I should have amputated the index and middle fingers. I, however, consented to bring the parts in apposition and retained them by sutures and adhesive straps; gave him a full dose of morphia to allay pain and induce sleep. The next day I found him suffering considerable pain and fever, and the hand considerably swollen; ordered an alterative with Dover's Powder internally, with cold water dressings to the hand.

May 6th—Patient more comfortable, and hand less swollen. Continued the cold water dressing, and a few grains of Pulv. Doveri at night to procure sleep.

May 10th—Removed the sutures and found that none of the surfaces had united by the first intention, but had commenced granulating with considerable purulent discharge. I enjoined strict cleanliness with dressings of simple cerate.

From this time the process of reparation went on regularly until the fingers were perfectly restored except a part of the last phalanx of the index finger.

